# Daily 30-min exposure to artificial gravity during 60 days of bed rest does not maintain aerobic exercise capacity but mitigates some deteriorations of muscle function: results from the AGBRESA RCT

**DOI:** 10.1007/s00421-021-04673-w

**Published:** 2021-04-03

**Authors:** Andreas Kramer, María Venegas-Carro, Jochen Zange, Wolfram Sies, Nicola A. Maffiuletti, Markus Gruber, Hans Degens, María Moreno-Villanueva, Edwin Mulder

**Affiliations:** 1 Human Performance Research Centre, Department of Sport Science, University of Konstanz, 78457 Konstanz, Germany; 2 Institute of Aerospace Medicine, German Aerospace Center (DLR), Cologne, Germany; 3 Human Performance Lab, Schulthess Clinic, Zurich, Switzerland; 4 Department of Life Sciences, Manchester Metropolitan University, Manchester, UK; 5 Institute of Sport Science and Innovations, Lithuanian Sports University, Kaunas, Lithuania

**Keywords:** Countermeasure, Astronaut training, Artificial gravity, Physical performance, Physical inactivity

## Abstract

**Purpose:**

Spaceflight impairs physical capacity. Here we assessed the protective effect of artificial gravity (AG) on aerobic exercise capacity and muscle function during bed rest, a spaceflight analogue.

**Methods:**

24 participants (33 ± 9 years, 175 ± 9 cm, 74 ± 10 kg, 8 women) were randomly allocated to one of three groups: continuous AG (cAG), intermittent AG (iAG) or control (CTRL). All participants were subjected to 60 days of six-degree head-down tilt bed rest, and subjects of the intervention groups completed 30 min of centrifugation per day: cAG continuously and iAG for 6 × 5 min, with an acceleration of 1*g* at the center of mass. Physical capacity was assessed before and after bed rest via maximal voluntary contractions, cycling spiroergometry, and countermovement jumps.

**Results:**

AG had no significant effect on aerobic exercise capacity, flexor muscle function and isometric knee extension strength or rate of force development (RFD). However, AG mitigated the effects of bed rest on jumping power (group * time interaction of the rmANOVA *p* < 0.001; iAG − 25%, cAG − 26%, CTRL − 33%), plantar flexion strength (group * time *p* = 0.003; iAG − 35%, cAG − 31%, CTRL − 48%) and plantar flexion RFD (group * time *p* = 0.020; iAG − 28%, cAG − 12%, CTRL − 40%). Women showed more pronounced losses than men in jumping power (*p* < 0.001) and knee extension strength (*p* = 0.010).

**Conclusion:**

The AG protocols were not suitable to maintain aerobic exercise capacity, probably due to the very low cardiorespiratory demand of this intervention. However, they mitigated some losses in muscle function, potentially due to the low-intensity muscle contractions during centrifugation used to avoid presyncope.

## Introduction

Without countermeasures, long-duration space missions—such as a mission to Mars—pose several hazards for astronaut health and performance, among others the deterioration of the cardiovascular and neuromuscular system, resulting in marked losses of strength, power, and endurance (Adams et al. 2003; Pavy-Le Traon et al. 2007; Rittweger et al. 2018; Williams et al. 2009). The lower extremities seem to be predominantly affected, which is consistent with the fact that they bear most of the bodyweight and will therefore be most affected by reduced weight-bearing activity (LeBlanc et al. 1992). In addition, the loss of plasma volume and the continuous cranial fluid shift have been associated with impaired orthostatic tolerance and the development of the Spaceflight-Associated Neuro-Ocular Syndrome (SANS) (Lee et al. 2018), respectively.

Several potential countermeasures for physical performance deteriorations caused by the lack of gravitational loading have already been tested in spaceflight analogues such as bed rest with head-down tilt (HDT). In a recent 60-day bed rest study, a high-intensity jump training program was successful in preventing a loss in bone mass, muscle mass, strength, and power, as well as aerobic exercise capacity, whereas the control group lost up to 50% (Kramer et al. 2017a, b, 2018). However, the effects induced by the cranial fluid shift and the loss of plasma volume were not completely prevented (Kramer et al. 2017b; Schoenrock et al. 2018). As most of the adverse spaceflight effects are the result of lacking gravitational forces, substituting the gravitational force with artificial gravity (AG), e.g., centrifugal force in a short-arm human centrifuge, could potentially attenuate all these effects. Some studies support this notion: in a recent review, Clément et al. (2016) provided an overview of the findings of short-arm centrifugation during bed rest and reported improved orthostatic tolerance, attenuated plasma volume loss, higher exercise capacity and less severe responses to head-up tilt, especially when the centrifugation was administered in an intermittent fashion (Linnarsson et al. 2015). In addition to the potential benefits for cardiovascular function, it has been suggested that centrifugation could also mitigate muscle atrophy, bone demineralization, and impairment of neuromuscular and sensorimotor coordination (Linnarsson et al. 2015; Young and Paloski 2007). However, these studies were too short to thoroughly examine the efficacy of short-arm centrifugation on neuromuscular and cardiovascular health and performance. Therefore, the space agencies wanted to determine the efficacy of AG during a long-term bed rest study, and assess whether administering AG in intermittent bouts rather than one continuous bout would be beneficial for AG acceptance, tolerability, orthostatic tolerance, and SANS.

The aim of this part of the bed rest study was to assess the efficacy of 30 min of AG per day as a countermeasure for the deconditioning effects induced by 60 days of bed rest on physical performance. We hypothesized that both AG protocols (continuous and intermittent) mitigate some of the expected alterations, attenuating the disuse-related losses in maximal oxygen uptake capacity and muscle function. As the two AG protocols did not differ in centrifugation duration or gravitational loading, we did not expect differences between the two AG groups with respect to the efficacy of the AG interventions on physical performance.

## Methods

### Study design

**Fig. 1:**
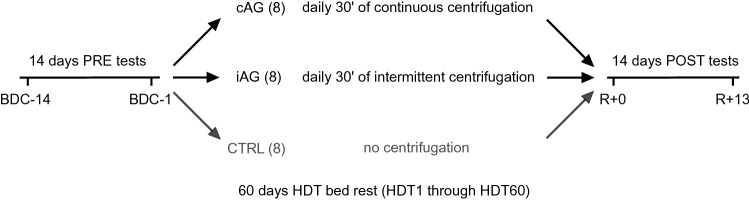
Study design. Prior to the bed rest phase, participants spent 14 days in the bed rest facility for familiarisation and baseline data collection (BDC-14 through BDC-1). In the morning of the first head-down tilt bed rest day (HDT1), participants were randomly assigned to one of the two intervention groups (cAG, 30 min of daily centrifugation during the 60 days of bed rest; iAG, 6 × 5 min of daily centrifugation interspersed with breaks of 3 min each) or the control group (CTRL). After the 60 days of HDT bed rest, participants were re-ambulated and stayed for an additional 14 days in the bed rest facility for measurements and recovery (*R* + 0 through *R* + 13)

This randomized controlled single-center, parallel-group trial (RCT) with balanced randomization (named AGBRESA) was conducted in 2019 at the: envihab facility of the German Aerospace Center (DLR) in Cologne, Germany. The study was split into two campaigns with 12 participants each. Each campaign consisted of 14 days of baseline data collection (BDC-14 through BDC-1), 60 days of HDT bed rest (HDT1 through HDT60), and 14 days of recovery (*R* + 0 through *R* + 13), see Fig. 1. The data reported in this article were recorded on the following days: BDC-10, BDC-5, BDC-3, *R* + 0 and *R* + 2 (see below for details).

### Subjects

Inclusion criteria were: healthy, age between 24 and 55 years, height 1.53–1.90 m, body mass index 19–30 kg/m^2^, non-smokers for at least 6 months prior to study start, being in possession of medical insurance. Exclusion criteria were: being vegetarian or vegan, requirements of prescription medication (including contraceptives), presence of substance abuse, criminal record, or health conditions that would preclude participation. These health conditions included existing or a history of cardiovascular dysfunction, musculoskeletal, ophthalmological, neurological and psychiatric conditions, metabolic or endocrine disturbances (e.g., diabetes mellitus), blood clotting, current or history of pulmonary disease, sleep and pain disorders, gastro-esophageal reflux, renal stones, infectious or inflammatory diseases. At the end of the second campaign, 24 participants had successfully completed the study. In the morning of HDT1, an envelope containing their pre-planned group assignment (balanced randomization, matched for sex, age and weight) was opened to reveal their assignment to either the intermittent centrifugation group (iAG, *n* = 8, age 34 ± 11 years, height 174 ± 11 cm, body mass 71 ± 5 kg, 3 women), the continuous centrifugation group (cAG, *n* = 8, age 32 ± 10 years, height 173 ± 8 cm, body mass 72 ± 10 kg, 3 women) or the control group (CTRL, *n* = 8, age 34 ± 8 years, height 177 ± 7 cm, body mass 79 ± 13 kg, 2 women). Except for a slight difference in height between cAG and CTRL (*p* = 0.03), no significant differences were observed between the three groups with respect to age, height and body mass. The second campaign started with 11 participants due to a last-minute drop-out. Shortly after the start of the study an additional 3 individuals were discharged from the study on medical grounds; the diagnoses were not related to the study and none exhibited clinical signs or symptoms during the screening process. To re-establish a full cohort of 12 participants during the second campaign, 4 additional participants were recruited and enrolled in the study. These 4 new subjects started 3 weeks after the other 8 participants and completed the entire study protocol. Before taking part in the study, all participants gave written informed consent to the experimental procedures, which were approved by the ethics committee of the Northern Rhine Medical Association (Ärztekammer Nordrhein, application No. 2018143) in Düsseldorf, Germany, as well as the Federal Office for Radiation Protection (Bundesamt für Strahlenschutz, application No. 22464/2018-074-R-G). Subjects received monetary compensation for participating in the study. The study is registered at the German Clinical Trials Register (No. DRKS00015677).

### Bed rest routine

During the bed rest period (starting at 9 a.m. on HDT1), all subjects maintained strict six-degree HDT bed rest for 24 h/day. All activities, including personal hygiene activities (bowel movement, showering, etc.) were performed in the HDT position. Previous studies identified that the usage of a pillow might confound results in the development of SANS (Laurie et al. 2019; Lawley et al. 2017). Therefore, the use of a pillow was prohibited once the subject entered the HDT phase of the study. Round-the-clock staff and video monitoring ensured compliance with the protocol. During the baseline and recovery phases (BDC and *R*), physical activity was restricted to free movement within the ward. Additional reconditioning sessions were administered post bed rest by physiotherapists. During the entire study, the subjects received a strictly standardized and controlled diet, with a daily water intake of 50 mL/kg body mass and an energy intake of 1.6 and 1.3 times the resting metabolic rate during the ambulatory phases and during HDT, respectively.

### Centrifugation

**Fig. 2:**
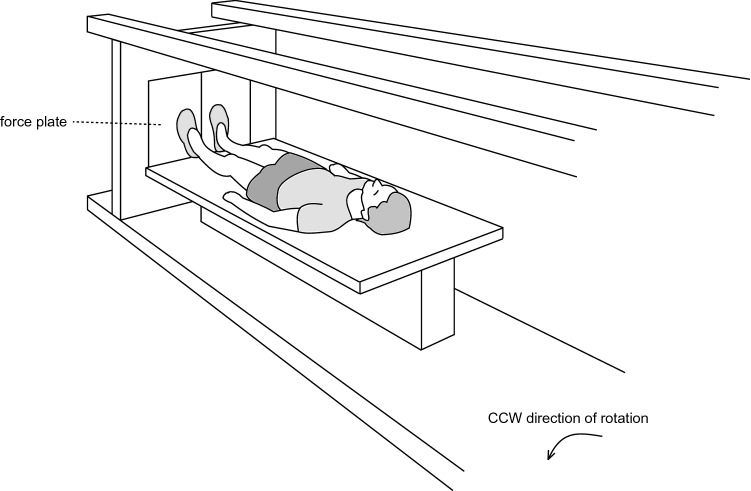
Centrifuge. For the artificial gravity interventions, a short-arm human centrifuge was used. Participants lay supine on a thin mattress on one arm of the centrifuge, feet pointing outwards. Rotational speed and the participant's position with respect to the centre of rotation were individually adjusted to achieve an acceleration of 1*g* at the estimated centre of mass and approximately 2*g* at the feet. The figure was first published in (Kramer et al. 2020b)

The DLR short-arm human centrifuge has a radius of 3.8 m [for a schematic representation of the centrifugation see Fig. 2, for more details on the system see previous publications using this centrifuge (Kramer et al. 2020a; Dreiner et al. 2020)]. The participant's position with respect to the center of rotation as well as the rotational speed of the centrifuge were adjusted to achieve an acceleration of 1*g* at the estimated center of mass and approximately 2*g* at the feet. All subjects were familiarized with the centrifugation once during medical screening and two additional times during the baseline phase (BDC-11 and BDC-4). Participants were instructed to stay in the supine position during centrifugation, to maintain the gaze fixed on a point, to avoid head movements and to contract exclusively the lower extremity muscles to avoid presyncopal symptoms. As there were considerable differences in centrifugation tolerance, this latter instruction resulted in some participants not contracting their muscles beyond normal background activity, while others were using the muscle pump almost constantly (Kramer et al. 2020b). Participants in the two intervention groups (cAG and iAG) completed one daily session of 30 min of centrifugation, either continuously for cAG (1 × 30 min) or intermittently with 6 × 5 min bouts interspersed with 3-min breaks for iAG. These centrifugation protocols were based on a previous short-term bed rest study (Rittweger et al. 2015) and aimed to assess benefits of and differences between the two protocols with respect to AG tolerance and SANS, as well as the potential of AG as a countermeasure for the deleterious effects of bed rest. All sessions were supervised by a medical doctor and were carried out in a horizontal position, i.e., at 0° HDT. Immediately prior to, and after the end of the centrifuge run, subjects were positioned in 6° HDT, whilst on the centrifuge. Rotation direction was changed from day to day so that every participant underwent half of the sessions in clockwise direction of rotation and half of the sessions in counterclockwise rotation.

### Aerobic exercise capacity

**Fig. 3:**
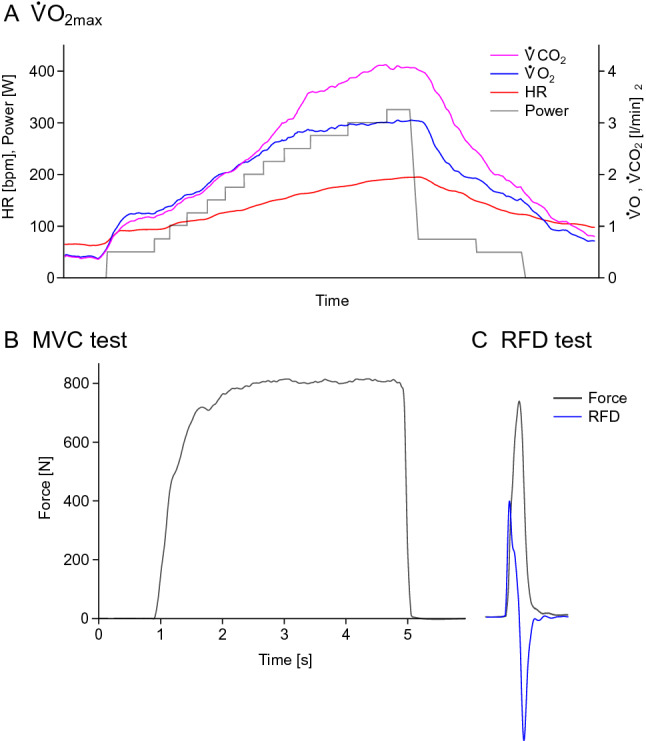
Exemplary data. **a** Data from one V̇O_2max_ step test on the cycle ergometer. Carbon dioxide is shown in pink, oxygen uptake in blue, heart rate in red, and power increments in grey. The test starts with 2 min of seated rest, followed by 3 min of warm-up at 50 W. Afterwards, the load is increased every minute in steps of 25 W until volitional exhaustion occurs (in this example at 325 W), followed by 4 min of active recovery (75 W and 50 W) and 4 min of passive recovery. Note that the figure shows breath-by-breath data, making the steps with higher load appear to be longer due to the increased breathing frequency. **b** Force data from one maximal contraction (plantar flexion) on the ankle ergometer. **c** Data from an explosive contraction (plantar flexion), with the force recording shown in grey and the first derivative of the force signal (for RFD calculation) plotted in blue

Maximal oxygen uptake (V̇O_2max_) during cycling was measured on a cycle ergometer (Lode, Groningen, The Netherlands), once before bed rest (BDC-3) and once after bed rest (*R* + 0). After an initial rest period of 2 min (while seated on the ergometer), subjects were instructed to start pedalling and maintain a cadence of approximately 80 rpm. The load was increased every minute in steps of 25 W (starting from 3 min at 50 W) until volitional exhaustion under strong verbal encouragement (Kramer et al. 2017a). The recovery period consisted of 4 min of low intensity cycling (2 min at 75 W, followed by 2 min at 50 W) and 4 min of passive rest (Fig. 3a). Subjects could, however, keep pedalling in the last 4 min at a low cadence, if they were hypotensive. Subjects were asked to rate their perceived exertion on a 6–20 point Borg scale. Breath-by-breath oxygen uptake and carbon dioxide emission were recorded using the Innocor system (Innovision, Odense, Denmark). Heart rate was continuously recorded via 12-lead ECG (Padsy, Medset Medizintechnik, Germany). Spiroergometry data were filtered by calculating the median of 5 breaths and subsequently the moving average over 30 s (Robergs et al. 2010). Afterwards, the peak values for the following parameters were extracted: absolute and relative V̇O_2_, heart rate, respiratory exchange ratio, and ergometer power. A test would be excluded from further analyses if the peak respiratory exchange ratio was below 1.10, the rating of perceived exertion on the Borg scale was below 17, or if there were inconsistencies between measurements before and after bed rest. This was, however, not the case for any of the tests. From the recovery period, the heart rate after the 4 min of active recovery (low intensity cycling) was extracted.

### Isometric muscle strength and rate of force development

Maximal voluntary isometric contraction (MVC, Fig. 3b) strength and rate of force development (RFD, Fig. 3c) were recorded unilaterally (right side) in custom-made knee and ankle ergometers for knee extension and plantar flexion. Subjects were initially familiarized with the equipment and testing procedures. They were measured twice before (BDC-10 and BDC-3) and once directly after bed rest (*R* + 0). Knee extension MVC and RFD were assessed in a sitting position with the hip and knee joints at 90° and 60° of flexion, respectively. Plantar flexion MVC and RFD were assessed in a sitting position with 90° at the hip, knee and ankle joints. The tests were preceded by a warm-up procedure consisting of six submaximal ramp-and-hold contractions. MVC tests (knee extension and plantar flexion) consisted of three repetitions of about 3 s, during which subjects were instructed to contract their muscles “as hard as possible” with a progressive force build-up. Rest periods of 2 and 5 min were interspersed between repetitions and between tests, respectively. RFD tests were performed after the respective MVC test, consisted of at least 10 repetitions separated by 20 s of rest and were conducted according to recent recommendations (Maffiuletti et al. 2016). Participants were instructed to contract their muscles “as fast and hard as possible” with particular emphasis on a fast increase in force, using short contractions (less than 1 s). Trials with unstable baseline, visible countermovement or a peak force of less than 80% of MVC were discarded.

Force data (analogue signals of the ergometer load cells) were amplified (Kistler 5006), sampled at 2 kHz and stored for later offline analysis. Analyses consisted of correcting possible offsets, followed by extraction of the maximal force for the MVC trials, and by calculating the first derivative, applying a 6th-order zero-lag Butterworth filter (cut-off frequency 15 Hz) and then extracting the maximal RFD for the RFD trials (Buckthorpe et al. 2012), respectively, via a Matlab script (Mathworks Inc, Natick, USA). For MVC, the maximum of the three trials was used for further statistical analyses, and for RFD, the average of the best three trials was retained (Maffiuletti et al. 2016).

### Isokinetic muscle strength

Maximal voluntary isokinetic strength was recorded with the Isomed 2000 system (D&R Ferstl GmbH, Hemau, Germany) for the following six tasks: unilaterally (right side) for knee extension, knee flexion, plantar flexion and dorsiflexion, as well as bilaterally for trunk extension and trunk flexion. Measurements were conducted once before (BDC-5) and once after bed rest (*R* + 2). Knee extension and knee flexion torque were assessed in a sitting position with the hips at 90°, knee range of motion was 20° to 95°, and the angular velocity was set at 60°/s. Plantar flexion and dorsiflexion torque were assessed in a supine position with hips and knees fully extended (0°); ankle range of motion was − 20° to 35°, and the angular velocity was set at 30°/s. Trunk flexion and extension torque was assessed in a sitting position; trunk range of motion was − 20° to 50°, and the angular velocity was set at 60°/s. Isokinetic tests were preceded by a warm-up procedure consisting of 5 min of cycling at 75 W and five submaximal isokinetic contractions. For each task, subjects completed three repetitions. Isokinetic torque data (analogue torque signals of the Isomed 2000) were first corrected for possible offsets (i.e., subtracting the average torque recorded at rest from the torque recorded during the contractions), then the maximal torque of each trial was extracted via a Matlab (Mathworks Inc, Natick, USA) script. Only the trial with the highest torque was retained.

### Jumping power

This test consisted of three maximal countermovement jumps (CMJ) on a force plate (Leonardo GRFP, Novotec medical GmbH, Pforzheim, Germany). Prior to the first test, all participants were shown and practiced the correct execution of the CMJ. They were asked to place their hands on the hips and “quickly drop to a half-squat position and then immediately jump as high as possible”. One minute of rest was given between each jump. The vertical ground reaction forces were recorded via a data acquisition unit (Power1401-3, CED, Cambridge, United Kingdom). Jump height was calculated based on the velocity at takeoff (antiderivative of the ground reaction force (GRF); jump height = (velocity at takeoff)^2^/2*g*), and jump power was calculated as the product of vertical GRF and velocity. Only the highest jump was retained.

### Statistics

Changes in response to bed rest were assessed on a per-protocol basis with repeated-measures analyses of variance (rmANOVA), using time (average of the two BDC measurements and *R* + 0) as a repeated measure and group (iAG, cAG, CTRL) as an inter-subject factor. In case Mauchly’s test of sphericity produced significant results, the Greenhouse–Geisser correction was applied. Sex differences of the bed-rest induced changes in physical performance normalized to baseline levels were assessed via two-tailed independent t-tests, as were potential differences between the groups at baseline with respect to age, height and weight. The analyses of the sex differences were performed separately instead of being included in the rmANOVA because baseline performance levels were different between men and women. Until HDT1, study personnel and participants were not aware of the group allocation (with the exception of the project manager and his deputy), and no strict measures were taken to blind outcome assessors and data analysts, even though most of them were unaware of group allocation. Analyses were executed with JASP 0.84 (JASP, University of Amsterdam, Netherlands). Group data are presented as means ± standard deviations (SD), and the level of significance was set to 0.05. Sample size estimations were based on the results of previous bed rest studies (Taibbi et al. 2016): with an effect size of 0.5, an alpha error of 0.05, and a power of 0.8, a sample size of 8 participants per group was needed.

## Results

### Aerobic exercise capacity

**Fig. 4:**
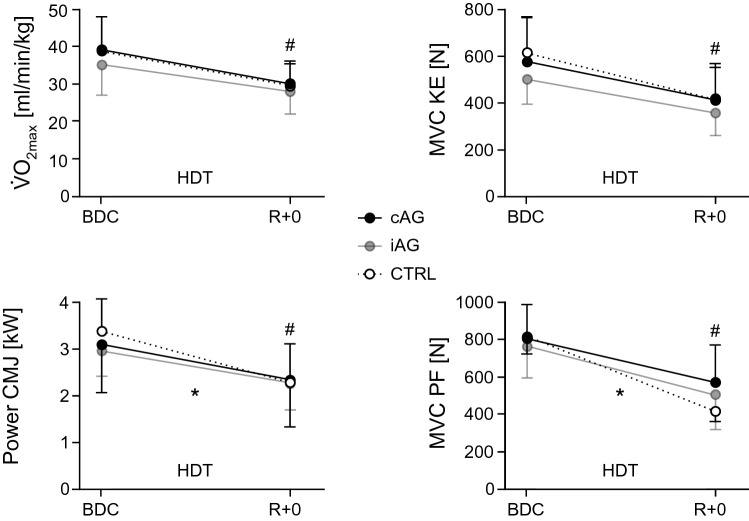
Maximal oxygen uptake (V̇O_2max_), peak power in the countermovement jump (CMJ), maximal isometric knee extension strength (MVC KE), and maximal isometric plantar flexion strength (MVC PF) before (BDC) and after bed rest (*R* + 0). Filled black circles represent the mean and standard deviation of the continuous artificial gravity group (cAG), filled grey circles the intermittent artificial gravity group (iAG), an open circles the control group (CTRL). An * symbol during HDT denotes a significant group by time interaction effect, a # symbol on top of the *R* + 0 data point denotes a significant main effect of time

There was no significant difference between groups with respect to the bed rest-induced loss of maximal oxygen uptake capacity V̇O_2max_ or any of the other parameters measured during the incremental exhaustive cycling test, i.e., there were no significant interactions, see Table 1 and Fig. 4.

**Table 1:** Aerobic exercise capacity, strength and power

	iAG BDC	iAG *R* + 0	cAG BDC	cAG *R* + 0	CTRL BDC	CTRL *R* + 0	Group * time Time
Spiroergometry							
rel. V̇O_2max_ [mL/(min kg)]	35 ± 8	28 ± 6	39 ± 9	30 ± 6	39 ± 9	29 ± 6	*p* = 0.31
		− 20 ± 5%		− 23 ± 5%		− 24 ± 8%	*p* < *0.001*
abs. V̇O_2max_ (L/min)	2.5 ± 0.7	2.0 ± 0.5	2.8 ± 0.8	2.1 ± 0.6	3.1 ± 0.7	2.3 ± 0.5	*p* = 0.23
		− 20 ± 5%		− 23 ± 5%		− 25 ± 7%	*p* < *0.001*
Peak power (W)	244 ± 65	181 ± 53	253 ± 80	181 ± 51	284 ± 48	206 ± 46	*p* = 0.41
		− 26 ± 5%		− 28 ± 6%		− 28 ± 6%	*p* < *0.001*
Peak HR (bpm)	180 ± 15	184 ± 17	189 ± 8	192 ± 14	189 ± 6	194 ± 9	*p* = 0.77
		+ 3 ± 3%		+ 1 ± 4%		+ 3 ± 3%	*p* = *0.003*
Recovery HR (bpm)	139 ± 18	148 ± 17	146 ± 11	168 ± 17	146 ± 15	160 ± 12	*p* = 0.21
		+ 9 ± 9%		+ 16 ± 4%		+ 12 ± 11%	*p* < *0.001*
Isom. strength							
MVC KE (N)	504 ± 107	356 ± 106	581 ± 190	417 ± 154	623 ± 146	411 ± 139	*p* = 0.11
		− 30 ± 11%		− 29 ± 10%		− 35 ± 10%	*p* < *0.001*
RFD KE (N/s)	4031 ± 999	3237 ± 1142	4128 ± 1555	3372 ± 1467	4540 ± 1324	3345 ± 1085	*p* = 0.41
		− 21 ± 19%		− 19 ± 16%		− 26 ± 12%	*p* < *0.001*
MVC PF (N)	767 ± 194	504 ± 177	808 ± 182	571 ± 200	820 ± 95	428 ± 65	*p* = *0.003*
		− 35 ± 10%		− 31 ± 15%		− 48 ± 6%	*p* < *0.001*
RFD PF (N/s)	4743 ± 1563	3431 ± 1103	4506 ± 2083	3971 ± 1991	4573 ± 1019	2670 ± 564	*p* = *0.02*
		− 28 ± 7%		− 12 ± 25%		− 40 ± 13%	*p* < *0.001*
Power CMJ (kW)	2.96 ± 0.69	2.26 ± 0.70	3.10 ± 1.05	2.39 ± 1.14	3.39 ± 0.69	2.34 ± 0.7	*p* < *0.001*
		− 25 ± 9%		− 26 ± 11%		− 33 ± 12%	*p* < *0.001*

	iAG BDC	iAG *R* + 2	cAG BDC	cAG *R* + 2	CTRL BDC	CTRL *R* + 2	Group * time Time
Isokin. strength							
Knee extension (Nm)	162 ± 45	123 ± 53	182 ± 70	144 ± 60	214 ± 36	141 ± 38	*p* = *0.03*
		− 25 ± 22%		− 22 ± 19%		− 35 ± 10%	*p* < *0.001*
Knee flexion (Nm)	99 ± 39	84 ± 35	100 ± 31	93 ± 37	107 ± 28	84 ± 22	*p* = 0.14
		− 15 ± 20%		− 10 ± 16%		− 21 ± 12%	*p* < *0.001*
Plantar flexion (Nm)	117 ± 32	95 ± 31	119 ± 43	95 ± 33	126 ± 37	79 ± 21	*p* = *0.04*
		− 19 ± 16%		− 18 ± 15%		− 36 ± 6%	*p* < *0.001*
Dorsiflexion (Nm)	31 ± 5	31 ± 7	35 ± 9	35 ± 10	37 ± 9	36 ± 9	*p* = 0.51
		− 2 ± 7%		− 2 ± 3%		− 3 ± 4%	*p* = *0.04*
Trunk extension (Nm)	180 ± 60	145 ± 66	171 ± 88	134 ± 67	169 ± 63	123 ± 54	*p* = 0.95
		− 22 ± 15%		− 20 ± 11%		− 26 ± 18%	*p* < *0.001*
Trunk flexion (Nm)	104 ± 36	108 ± 43	119 ± 43	111 ± 45	121 ± 26	110 ± 20	*p* = 0.36
		+ 2 ± 12		− 7 ± 13%		− 7 ± 14	*p* = 0.29

Results of the cycling spiroergometry test, the isometric maximal voluntary contraction (MVC) and rate of force development (RFD) tests, the countermovement jump (CMJ) test and the isokinetic strength tests, separately for the intervention groups (iAG and cAG) and the control group (CTRL), once during baseline (BDC: BDC-3 for the spiroergometry, average of BDC-10 and BDC-3 for the isometric strength tests and BDC-5 for the isokinetic strength tests) and once directly after bed rest (*R* + 0 and *R* + 2, respectively). The percent values reflect the averaged individual changes observed after bed rest compared to baseline. The last column contains the results of the rmANOVA (group * time interaction effect and main effect of time)

Significant p-values (i.e., all *p*-values smaller than 0.05) are set in italics

### Maximal strength

Maximal isometric and isokinetic extension strength was significantly decreased in all groups after bed rest, with less pronounced declines in the countermeasure groups, especially for plantar flexion, where the group by time interaction effect reached the level of significance, see Table 1 and Fig. 4. Maximal isokinetic strength of the lower extremity flexor muscles was affected to a lesser extent by bed rest compared to lower extremity extensors, and maximal trunk flexor strength did not even change significantly in response to bed rest.

### RFD

Maximal RFD showed a similar pattern as the maximal strength, i.e., bed rest-induced losses in RFD were comparable for knee extension across groups (no interaction effect), but smaller for plantar flexion RFD in the countermeasure groups compared to the control group (significant group by time interaction effect), see Table 1.

### Power

Jump power recorded during the CMJs significantly decreased in all three groups, but to a lesser extent in the two AG groups (significant group by time interaction effect, see Table 1 and Fig. 4).

### Sex differences

For the analysis of potential sex differences in response to bed rest, data from all three groups were pooled, thus allowing a comparison between the 16 male and 8 female volunteers. For some physical performance parameters, there was no significant difference between men and women—e.g., V̇O_2max_ or plantar flexion RFD—but in general, physical performance was more affected by bed rest in women than in men, and significantly so for peak jump power and maximal knee extensor strength, see Table 2.

**Table 2:** Changes physical performance men–women

	Men BDC	Men *R* + 0	Women BDC	Women *R* + 0	*t*-test
rel. V̇O_2max_ [mL/(min kg)]	42 ± 8	35 ± 5	30 ± 4	23 ± 2	*p* = 0.46
		− 23 ± 6%		− 21 ± 7%	
MVC knee ext (N)	643 ± 103	456 ± 91	403 ± 53	232 ± 24	*p* = *0.01*
		− 29 ± 9%		− 41 ± 11%	
RFD knee ext (N/s)	4896 ± 878	3920 ± 782	2908 ± 820	1935 ± 577	*p* = 0.20
		− 19 ± 13%		− 29 ± 20%	
MVC plantarflex (N)	886 ± 92	578 ± 135	624 ± 104	347 ± 87	*p* = 0.08
		− 35 ± 13%		− 44 ± 9%	
RFD plantarflex (N/s)	5379 ± 1248	3908 ± 1373	3065 ± 662	2255 ± 594	*p* = 0.93
		− 27 ± 22%		− 26 ± 16%	
Power CMJ (kW)	3.6 ± 0.5	2.9 ± 0.6	2.2 ± 0.3	1.3 ± 0.2	*p* < *0.001*
		− 22 ± 6%		− 39 ± 9%	

Changes in physical performance after the end of the bed rest phase (*R* + 0) compared to the baseline recordings before the start of the bed rest phase (BDC), separately for male and female participants (pooled data from all three groups cAG, iAG and CTRL). The last column contains the results of the two-tailed independent *t*-tests comparing the changes observed for the men to the changes observed for the women (averaged individual changes)

Significant p-values (i.e., all *p*-values smaller than 0.05) are set in italics

## Discussion

Overall, the AG interventions had no effect on the loss of aerobic exercise capacity observed after 60 days of bed rest. However, AG mitigated some of the losses observed for calf muscle function, dynamic knee extension strength and jumping power. Unexpectedly, some aspects of physical performance were more affected in women than in men, although this needs to be verified with larger sample sizes.

### Efficacy of AG for maintaining physical performance

As AG provides both loading and a caudal fluid shift, centrifugation was tested with the expectation that it would attenuate the bed rest-induced decrements in both the cardiovascular and musculoskeletal system, as suggested by short-term bed rest studies (Caiozzo et al. 2009; Stenger et al. 2012). However, the present study showed that cardiovascular exercise capacity (V̇O_2max_) could not be maintained by simply exposing subjects to 30 min of AG per day on a short-arm human centrifuge. Furthermore, we observed that recovery from the exhaustive cycling protocol was severely impaired in all groups after bed rest, as evidenced by the increased heart rate after the end of the test. We had already reported that the two AG interventions could not prevent an increase in resting heart rate after bed rest either (Kramer et al. 2020b). Furthermore, the analysis of oxygen uptake and muscle activity recordings of the daily 30 min centrifugation sessions had revealed that oxygen demand during centrifugation remained at resting levels and that muscle activity of the lower extremity muscles was rather low for most participants of the present study, with a preference for calf muscle activation (Kramer et al. 2020b). Thus, while the AG countermeasure had no significant impact on measures of cardiovascular function, the small protective effect on muscle function, particularly for calf muscles, may be attributed to the preferential calf muscle activation during centrifugation. This might also explain the mitigating effect of centrifugation on jumping power, but this is hard to pinpoint, as all lower extremity extensor muscles will contribute to jumping power, and dynamic knee extension strength was also less affected by bed rest in the AG groups compared to the control group. In any case, it is interesting to note that the AG countermeasure mitigated some deteriorations of muscle function despite its very low intensity. From an applied perspective, the partially protective effect of centrifugation on muscle function—even though statistically significant—does not seem to be large enough to justify the use of centrifugation alone as a countermeasure for the deleterious effects of microgravity on physical performance.

Lower extremity flexion strength was less affected compared to lower extremity extension strength, with dorsiflexion being hardly affected at all. This has been reported previously (Lee et al. 2014) and has been attributed to the higher susceptibility of “antigravity” muscles to gravitational unloading during bed rest or spaceflight (LeBlanc et al. 1992). The analyses of maximal trunk strength revealed a similar result: bed rest did not significantly affect trunk flexion strength, whereas trunk extension strength decreased by more than 20% across groups. To our knowledge, trunk strength has never been reported in previous bed rest studies. Our results are, however, consistent with results from magnetic resonance imaging findings obtained in a previous 56-day bed rest study, where the multifidus muscle, which stabilizes the spine and can act as a trunk extensor muscle, showed significant atrophy in response to bed rest, whereas abdominal (flexor) muscles showed no changes (Hides et al. 2007).

In cross-sectional studies, it has been reported that with increasing age RFD as well as muscle power decline faster than muscle strength (Korhonen et al. 2006; Skelton et al. 1994). As the performance decline during aging is usually the result of a combination of aging per se and decreased physical activity, it is possible that physical inactivity during bed rest would likewise result in a larger decrease of power and RFD than strength. This was, however, not observed in the current study; if anything, RFD and jumping power were slightly less affected than maximal strength, which might be an effect of a fibre type shift towards faster subtypes (Trappe et al. 2004; Borina et al. 2010), as well as increased shortening velocities of type I fibres that have been reported after previous bed rest studies (Widrick et al. 1997).

The differences between the two AG countermeasures were not analysed in more detail, as differences were expected mainly in countermeasure tolerance, SANS and orthostatic tolerance. Nevertheless, the mean changes in physical performance in response to bed rest were very similar in the two AG groups, i.e., the 1 × 30 min continuous protocol and the 6 × 5 min intermittent protocol seem to have a similar effect (or lack thereof) on physical performance.

### Sex-related differences

There seemed to be a trend for more pronounced bed rest-induced losses in women than in men for at least some parameters of physical performance, particularly in knee extension strength and power. Admittedly, the data from all subgroups were pooled, resulting in a comparison of 16 male and 8 female participants (average age 34 ± 8 and 33 ± 10 years, respectively), and even though the similar proportion of women in the three groups and the limited effect of AG justify the pooling, it limits the conclusions that can be drawn. Despite this shortcoming, it is an interesting observation that women seem to have more pronounced losses in some parameters than men, even though for women the values before bed rest were lower than for men. Usually, participants with the higher absolute values tend to lose more in response to physical inactivity or detraining, both in absolute and relative numbers (Ried-Larsen et al. 2017; Hughes et al. 2001; Forrest et al. 2007). The reason for the apparently higher loss observed in this study for women despite lower absolute baseline performance values is not entirely clear. It is difficult to compare our results to previous studies assessing sex-related differences during bed rest or spaceflight, as past bed rest studies have been conducted primarily with men only, and spaceflight studies usually comprise few women [less than 20% of the astronauts and cosmonauts in the last decades were women (Mark et al. 2014)]. The few studies that did compare physical capacity between men and women after bed rest or spaceflight reported no significant sex-related differences (Convertino et al. 1977; LeBlanc et al. 1995). Considering the high inter-individual variability observed in response to bed rest and spaceflight (Mark et al. 2014), studies with larger sample sizes and a higher proportion of women are required to determine if, how and why women respond differently than men (Harm et al. 2001).

### Comparison to previous bed rest studies

The loss in physical capacity observed in the control group is similar to what has been reported in previous bed rest studies of similar duration (Buehring et al. 2011; Kramer et al. 2017a), and also corresponds with the time-course of performance loss observed in bed rest studies with different durations (Capelli et al. 2006). The efficacy of AG as a countermeasure in that respect does not compare that well, however. For instance, the high-intensity jump training used in the 60-day RSL bed rest study prevented bed rest-induced decreases in muscle mass, strength and power, as well as V̇O_2max_ (Kramer et al. 2017a, 2018), even though the effective training duration was only few minutes per day. Granted, other exercise countermeasures were not able to fully prevent the deterioration in physical performance either (Alkner and Tesch 2004; Lee et al. 2014), and non-exercise countermeasures such as increased protein intake showed no protective effect at all (Lee et al. 2014; Schneider et al. 2009). However, given the positive tendencies reported after short-term AG bed rest studies, a higher efficacy was expected in the present study. For example, the 5-day BR-AG1 bed rest study with AG as a countermeasure showed positive effects on orthostatic tolerance (Linnarsson et al. 2015), and another 21-day bed rest study reported positive effects for muscle, bone and the cardiovascular system (Young and Paloski 2007; Stenger et al. 2012). The AG protocol used in the 5-day study was similar to the one used in the present AGBRESA study, but the 21-day study used 60 min rather than 30 min of AG per day. Therefore, it is possible that longer exposure to AG or higher g-levels could increase the efficacy of AG. Another perhaps more efficient approach to render AG more effective is its combination with exercise. Indeed, AG combined with stationary cycling has already been tested during several short-term bed rest studies, showing partial effectiveness for cardiac function (Yang et al. 2011), plasma volume, orthostatic tolerance and resting heart rate (Iwase 2005), as well as stroke volume and V̇O_2max_ (Katayama et al. 2004). However, the promising observations from these short-term bed rest studies—which seem to be limited to cardiorespiratory effects, with minor effects on muscle and bone (Beller et al. 2011)—have yet to be verified in long-term studies. Different types of exercise that target not only the cardiovascular system but also muscle and bone would probably be more suitable, for example the high-intensity jump training that proved to be very effective in maintaining muscle, bone and cardiovascular health in a previous long-term bed rest study (Kramer et al. 2017a). It was already demonstrated that jumping whilst being centrifuged is possible, albeit with reduced peak ground reaction forces and changes in the movement pattern that might lower the countermeasure efficacy (Dreiner et al. 2020; Kramer et al. 2020a; Frett et al. 2020). Hence, future studies are needed to (a) determine those exercise regimes that could be combined with AG exposure, and (b) disentangle the effects observed in studies combining centrifugation and exercise by comparing the effects of the combined intervention to the effects of exercise alone. If the positive effects are primarily due to exercise and not centrifugation, these exercise regimes are perhaps easier, safer and more efficient if performed without AG.

### Limitations

As it is usually the case with bed rest or astronaut studies, the sample size in the present bed rest study was substantial for such a type of study, but still rather small. This limits the possibility of valid subgroup analyses, with inter-individual differences sometimes larger than the between-group differences. Therefore, we only reported the results of the omnibus tests and refrained from performing more in-depth subgroup analyses such as post-hoc tests.

## Conclusion

Daily 30-min exposure to AG elicited by short-arm centrifugation was not effective for maintaining aerobic exercise capacity, probably due to the very low cardiorespiratory demand of this intervention. Despite the observation that the combination of centrifugation and low-intensity muscle contractions during centrifugation mitigated some of the losses in muscle function, AG would have to be combined with adequate exercise protocols before potentially being considered as an integral countermeasure for the deleterious effects of microgravity on physical performance losses.

## Abbreviations

AG: Artificial gravity
BDC: Baseline data collection
cAG: Continuous artificial gravity group
CMJ: Countermovement jump
CTRL: Control group
DLR: Deutsches Zentrum für Luft- und Raumfahrt, German Aerospace Center
*g*: Earth’s gravitational acceleration
GRF: Ground reaction force
HDT: Head-down tilt
HR: Heart rate
iAG: Intermittent artificial gravity group
MVC: Maximum voluntary contraction
RCT: Randomized controlled trial
RFD: Rate of force development
rmANOVA: Repeated measures analysis of variance
SANS: Spaceflight-Associated Neuro-Ocular Syndrome
V̇O_2max_: Maximal oxygen uptake
